# The Plague of Consumption

**Published:** 1895-03-30

**Authors:** John Gordon Dill

**Affiliations:** Assistant Physician to the Sussex County Hospital


### MEDICAL PROGRESS AND HOSPITAL CLINICS.

[*The Editor will be glad to receive offers of co-operation and contributions from members of the profession. All letters should be addressed to* THE EDITOR, THE LODGE, PORCHESTER SQUARE, LONDON, W.]

### THE PLAGUE OF CONSUMPTION.

By JOHN GORDON DILL, M.A., M.D., Assistant Physician to the Sussex County Hospital.

TUBERCULOUS disease, of which consumption is the most frequent manifestation, exists all over the world. There is no country, climate, or race which is free from its ravages, and it causes the death of about one seventh of mankind. We in England are less afflicted by it than some other nations, but of the half million deaths which occur among us every year, between sixty and seven thousand, viz., about one-eighth of the total number, are due to this disease in its various forms.

If this plague of consumption visited us annually in an epidemic form, and carried off its sixty thousand victims in the course of a few weeks, it would arouse a panic through the length and breadth of the land, the most stringent measures of precaution, disinfection, and isolation would be insisted upon, and every effort would be made to stay its progress; but so insidious is its attack, so gradual usually its advances, so often undetected its first symptoms, that we have hitherto been content to accept it as the inevitable, and with an apathy which, with our present knowledge, is little short of criminal, to allow the seeds of poison to be sown unchecked, and, from want of timely warning, to permit the unfortunate sufferers in their ignorance to be a source of danger to those nearest and dearest to them, as well as to the whole community. It is only within the last few years that even the crudest and most inefficient attempts have been made to arrest the spread of the disease, not indeed, until 1882, when Koch published his classical researches upon the life history and habits of the tubercle bacillus. Its infective properties have been amply demonstrated by direct experiment, and it is found that it may make its way into the body of its host either by inoculation through a breach of the skin or mucous membrane, or by ingestion with the food into the alimentary canal, or by inhalation into the respiratory apparatus. In all probability the relative frequency of these three methods of infection is inversely in the order named, the last being by far the commonest.

When once the tubercle bacillus has found a resting-place in the body of its host, its presence and development give rise to irritation and consequent changes in the tissues surrounding it, and finally lead to their destruction. If this process has taken place in the lung, the diseased tissue is gradually coughed up with other inflammatory products, but by this time the bacilli have multiplied enormously, and myriads of them are contained in the expectorated matter. It has been calculated, indeed, that one consumptive patient may distribute broadcast no less than twenty millions of bacilli during twenty-four hours. The bacilli thus set free are unable, as far as we know, to multiply outside the body except under certain artificial conditions, but they retain their vitality for an extraordinary time, and were found by Koch to be still virulent and infective in the dust of dried-up sputum after six months. What wonder is it that this poison is ubiquitous? It has been found, in fact, in an active condition in the dust of the air, and in the dust of the walls of the dwellings of consumptives; and there can be no doubt that we, at any rate, who live in towns are constantly exposed to its deadly infection.

What is the dose of poison that will engender the disease in a healthy person remains yet to be determined. Undoubtedly there are provisions in a healthy organisation for ejecting some of the bacilli and killing others, yet there are many among us who from inheritance, from the weakening effects of certain diseases, from ill-nutrition and insanitary surroundings, from the results of inflammation, irritation, and disease of the respiratory apparatus, or from deficient expansion of the lung, present a peculiarly fertile soil to the bacillus, and offer little or no resistance to its attack.

It is still almost universally imagined that the certain diagnosis of consumption is equivalent to a sentence of death, but this is not the case. Under suitable conditions the disease is by no means incurable, and in fact great numbers suffer from attacks of pulmonary tuberculosis and recover without its being even suspected. This was recognised more than fifty years ago by a French physician who discovered evidence of former tuberculosis of the lungs in 51 per cent. of the autopsies of patients who had died of other diseases; and Dr. Harris, in an interesting paper published in 1889, states that he found the same in 38·84 per cent. of the autopsies at the Manchester Royal Infirmary. Dr. Theodore Williams, in his recently published Lumleian lectures, gives some striking statistics of his cases, in which the proportion of cures and of arrest of disease is very great.

At the same time, prevention is better than cure, and in the present state of our knowledge of the disease there is no reason, theoretically, that it should not be completely stamped out from among us. The analogy between tubercle and leprosy has been often insisted upon; their bacilli are almost indistinguishable, they are equally independent of climate or race, and their modes of growth and of development are very similar, yet although at one time these islands were infested with leprosy, the efforts of our forefathers have completely expelled it. This was effected, it is true, by the most rigorous leprosy laws, entailing a degree of hardship and isolation upon the unfortunate lepers, which would not be necessary in the case of consumptives. The evidence of direct contagion from a tuberculous to a healthy person is very slight. The expectoration of consumptives is almost certainly the chief channel through which the poison is disseminated, and could we insure the effectual and prompt disinfection of all tuberculous sputa, it is not too much perhaps to assume that in a few years consumption would be a comparatively rare disease; but at present countless millions of active bacilli are let loose daily upon our streets and in our dwellings, without an attempt to render them innocuous.

A more general public knowledge of the infective nature of tuberculosis, that it does not arise *de novo*, and that it is in the highest degree preventable, would undoubtedly do much to change all this, and a great deal, of course, lies in the hands of medical men.

Among the rich and the well-to-do there appears to be nothing to prevent a consumptive patient living at home without danger to the healthy members of his family, provided always that certain precautionary measures are rigorously observed; it is among the poor and the labouring classes that the chief danger lies. As an illustration of this a case may be taken which, as a type, must be a matter of daily experience to the clergy and those who work among the poor, and to every physician in the out-patient department of a general hospital.

A labouring man, aged 28, with a wife and five young children, is attacked by consumption. As time goes on his work becomes intermittent, and is finally brought to an abrupt conclusion by an attack of hæmorrhage, or some other complication. Meanwhile the little home has been broken up, and the club subscription has been long unpaid. The family are now living together in one room, and the wife tries to support her invalid husband and the children by what work she can get, aided by parish relief. The district visitors and the clergy help with gifts of nourishing food, but this usually goes to the children, and the invalid father after growing gradually weaker during a few months, at length succumbs to the disease. The widow cannot afford to leave the infected room, and indeed she probably clings to it, as it contains the relics of the former home. In the course of time she and one of the children develop the disease and die, and the survivors are received into the workhouse. Then the room is let to another family who move into it without disinfection, or perhaps even cleansing.

Now to deal effectually with cases of this kind, special hospitals for consumption built in healthy open spaces near every large centre of population, are an absolute necessity. Patients in an early stage of the disease would have an infinitely better chance of being cured in an institution of this kind, where they would have good food, hygienic surroundings, and no danger of constant re-infection from the poisonous dust of their own dwellings, and where they could be taught the necessary measures of prevention should they sufficiently recover to return to work. Many useful lives would in this way be saved; but if, on the other hand, they went from bad to worse, they would have a comfortable asylum where they could be visited by their relatives and friends, and they would be no longer a source of danger either to them or to the rest of the community.

In the Middle Ages leper houses were scattered all over the kingdom, but to-day the special hospitals for consumption can almost be counted upon the fingers. What nobler object can there be for the charitable than the establishment of such institutions, until their existence is recognised as an absolute necessity of civilisation?

But further than this, if we are ever to stamp out consumption, stringent legislation is undoubtedly required to prevent the sale of tuberculous milk and meat, to deal with the occurrences of tuberculosis in animals, to ensure the due sanitation and disinfection of the dwellings of consumptives, to enforce the compulsory notification of the disease, and to control the disposal of the bodies of its victims.

All this will come in time, but no reform can be undertaken successfully without a wide publicity and a popular demand. Let those, therefore, who know the dangers of the poison so widely spread around us, and the possibilities of controlling it, shake off their apathy, and enlighten others.

We are now losing the flower of our nation from this fell disease. Many of the brightest, the most promising, the most beautiful in body and mind are untimely cut off upon the threshold of life.

Every step in advance towards the prevention of tuberculosis will tend to the improvement of our race, will lessen the poverty, misery, squalor, and crime in our midst, and will lead to the greater happiness and safety of our children, and of unborn generations of their descendants.

